# Availability of telehealth-based services at syringe services programs under the COVID-19 Public Health Emergency

**DOI:** 10.1186/s12954-023-00861-3

**Published:** 2023-09-02

**Authors:** Tyler S. Bartholomew, Hansel E. Tookes, Teresa A. Chueng, Ricky N. Bluthenthal, Lynn D. Wenger, Alex H. Kral, Barrot H. Lambdin

**Affiliations:** 1 University of Miami, 1120 NW 14th St #860, Miami, FL 33136 USA; 2 University of Southern California, Los Angeles, CA USA; 3 RTI International, Berkeley, CA USA

**Keywords:** Telehealth, Syringe services programs, Harm reduction, COVID, People who inject drugs

## Abstract

**Introduction:**

The expanded capacity of syringe services programs (SSPs) in the USA to integrate telehealth services was largely related to flexibility of buprenorphine prescription in response to the COVID-19 pandemic. SSPs demonstrated the potential of using telehealth to reach participants with both medical and non-medical services. The present study examines the implementation of medical and non-medical telehealth-based health services in 2020 at SSPs in the USA and organizational characteristics associated with adopting specific telehealth services.

**Methods:**

We administered a cross-sectional survey among all known SSPs operating in the USA as of 2021. The two primary study outcomes were (1) implementation of medical telehealth and (2) implementation of non-medical telehealth in 2020. Medical services included HIV counseling/care, hepatitis C virus (HCV) counseling/care, and buprenorphine. Non-medical services included wellbeing/check-ins, overdose prevention training, health navigation, harm reduction and psychological counseling. Bivariate and multivariable mixed effects logistic regression models were used to directly estimate the odds ratio associated with organizational characteristics on the implementation of telehealth-based health services.

**Results:**

Thirty percent of programs (*n* = 290) reported implementing telehealth-based health services. In multivariable logistic regression models, community-based organization SSPs had higher odds of implementing medical (aOR = 4.69, 95% CI [1.96, 11.19]) and non-medical (aOR = 2.18, 95% CI [1.10, 4.31]) health services compared to public health department SSPs. SSPs that received governmental funding had higher odds of implementing medical services via telehealth (aOR = 2.45, 95% CI [1.35, 4.47]) compared to programs without governmental funding.

**Conclusion:**

Community-based organization SSPs and those with government funding had the highest odds of telehealth implementation in response to the COVID-19 Public Health Emergency. Federal, state, and local governments must increase funding for low-barrier venues like SSPs to support telehealth implementation to serve the needs of people who use drugs.

## Introduction

The overdose mortality crisis in the USA culminated in 106,669 drug overdose deaths in 2021 [1, 2]. Social instability, interruption of drug markets, and adulteration of the drug supply exacerbated by the COVID-19 pandemic dramatically shifted the risk landscape for people who use drugs [3]. At the programmatic level, syringe services programs (SSPs) felt the impact on their ability to deliver interventions for people who inject drugs (PWID) and link people to substance use disorder (SUD) treatment [4–8]. Together, these dynamics increased the risk of HIV and hepatitis C virus (HCV) outbreaks and synergistically contributed to the highest number of overdose fatalities occurring in a 12-month period in the USA to date [1] .

SSPs are evidence-based, indispensable public health programs that undertake all four pillars (Diagnose, Treat, Prevent, and Respond) of the Ending the HIV Epidemic Initiative [9]. Beyond provision of sterile drug equipment and naloxone, an SSP can also provide a comprehensive, culturally competent array of infectious disease and SUD services for PWID. This includes harm reduction counseling, psychological evaluation, HIV prevention, antiretroviral therapy, HCV treatment, and medications for opioid use disorder (MOUD) [10, 11] . Between 2015 and 2018, the number of SSPs in the USA increased rapidly [12, 13], growing to over 400 organizations [5]. SSPs have proved to be invaluable in reaching individuals who historically have had inadequate access to critical health interventions, including SUD treatment.

Since the start of the COVID-19 pandemic, telehealth has been implemented and scaled-up to reduce the risk of COVID-19 transmission [14]. In response to the COVID-19 Public Health Emergency, federal agencies adapted policies to allow for the use of telehealth across various health settings. Of note, the in-person requirements for buprenorphine prescription under the 2008 Ryan Haight Act were suspended and the Centers for Medicare & Medicaid Services rapidly instituted temporary rules and waivers to expand the scope of Medicare telehealth services in March 2020 [15–17]. This facilitated a rapid, large-scale implementation of telehealth programs, particularly in the treatment of mental health and SUD [17] across traditional and non-traditional settings, including SSPs [11, 18–23].

After the waiver of the Ryan Haight Act, our previous cross-sectional study found that a quarter of SSPs in the USA reported the implementation of telehealth buprenorphine services [20]. Results also revealed that certain SSP organizational characteristics (i.e., larger budgets and community-based SSPs) were associated with higher likelihood of telehealth buprenorphine implementation [20]. While the substantial rise of telehealth encounters at SSPs has enabled receipt of low-barrier buprenorphine services, it is unknown to what extent SSPs programmatically adapted other telehealth services to meet the medical and non-medical needs of their participants at the beginning of the COVID-19 pandemic.

The expanded capacity of SSPs in the USA to integrate telehealth services was largely related to flexibility of MOUD prescription in response to the COVID-19 pandemic [15, 20]. But, SSPs also demonstrated the potential of using telehealth modalities to reach participants with other medical and non-medical services. Improved understanding of how general telehealth services were adapted across the USA in 2020 could facilitate future programmatic planning and implementation at SSPs, while also informing future policy. Enhanced accessibility and health equity for PWID, a community who have historically received disproportionately inadequate and fragmented care, could be achieved. Fortunately, in February 2023, the Drug Enforcement Agency proposed to make permanent changes to the rules adopted during the COVID-19 Public Health Emergency for telehealth flexibilities [24] . The present study is thus a timely examination of the medical and non-medical telehealth-based health services implemented by SSPs in the USA in 2020 and organizational characteristics associated with adopting specific telehealth services.

## Methods

### Human subjects

This study was reviewed and approved by the Office of Research Protection at RTI International’s Institutional Review Board.

### Study setting and design

We administered a cross-sectional survey among all known SSPs operating in the USA as of 2021. In collaboration with the North American Syringe Exchange Network (NASEN), we built a dataset of SSPs from multiple sources, including NASEN’s online directory and buyers’ program, regional networks of SSPs, public health department websites, social media platforms, webinars, and conferences. Our team proactively contacted SSPs to understand whether the SSPs were currently operating, and to identify updated contact information.

### Data collection procedures

SSPs in the USA were recruited to complete an online survey (average completion took 35 min) between February and June 2021. SSPs within our dataset were sent an email invitation to complete the survey. Organizational directors were contacted up to two additional times via email with reminders to complete the survey. If programs were non-responsive, the study team reached out to each program individually via email and/or phone call. A financial incentive ($75 honorarium) was provided to each program that completed the survey. Survey responses were collected and stored using the Voxco platform (Voxco, Montreal, Canada).

### Measures

#### Independent variables

Variables of interest for this present analysis included: type of SSP (public health department (PHD-SSP) vs. community-based organization (CBO-SSP)), census region where the SSP was located (West, South, Northeast, Midwest), annual budget (rounded to the nearest dollar), types of funding to support the SSP (governmental vs. no governmental), and number of different sources of funding (0, 1, 2, 3,  >  = 4).

### Primary outcomes

There were two primary study outcomes of interest for this present analysis: (1) whether an SSP had newly implemented a medical, telehealth-based health service in 2020 and (2) whether an SSP had newly implemented a non-medical, telehealth-based health service in 2020. Medical services included HIV status counseling, HIV medical care, HCV status counseling, HCV medical care, and buprenorphine prescription. Whether or not status counseling included self-administered rapid tests was not queried. Non-medical services included wellbeing/check-ins, overdose prevention training, health navigation, harm reduction counseling, and psychological counseling. Telehealth was defined as providing a health-related service or information for SSP participants via phone, tablet, or computer. Data were collected and analyzed as a binary outcome (yes/no).

### Statistical analysis

Frequency distributions and percentages were calculated for categorical variables and median and interquartile ranges were calculated for continuous variables to describe the overall characteristics of the sample. Bivariate and multivariable mixed effects logistic regression models were used to directly estimate the odds ratio associated with organizational characteristics on the implementation of telehealth-based health services. The census division where the SSP operated (South Atlantic, Middle Atlantic, New England, East North Central, East South Central, West South Central, West North Central, Mountain, and Pacific) was included in the adjusted models as a random effect to account for within cluster correlation. Results were reported as adjusted odds ratios (aOR) with corresponding 95% confidence intervals (CIs). All analyses were performed using SAS 9.4. statistical software (SAS Institute, Cary, North Carolina, USA) and significance level was set at an alpha of 0.05.

## Results

Of the 431 operating SSPs, 324 completed the survey. We excluded 34 programs from the analytic sample due to missing data regarding telehealth-based health service implementation. The final analytic sample was comprised of 290 SSPs. A majority of programs were CBO-SSPs (56.7%), but a majority also reported received some form of governmental funding (73.1%; Table 1). In addition, almost half were operating in the West census region (46.2%). About one-third (30.0%) of programs reported implementing a telehealth-based health service in 2020; specifically, 23.1% reported implementing a medical service and 27.9% reported implementing a non-medical service via telehealth. The most common telehealth-based interventions reported were harm reduction counseling (21.7%), overdose prevention training (19.7%), and health navigation/case management (19.0%), which are notably all non-medical services. Among medical services, specifically, the most common telehealth-based interventions reported were buprenorphine/Suboxone (16.9%), HIV status counseling (10.7%), and HCV status counseling (10.3%).

**Table 1:** Descriptive statistics of SSPs (*N* = 290)

Characteristics	Total *N* = 290 (%)
*Program type*	
CBO-SSP	161 (56.7)
PHD-SSP	123 (43.3)
*Census region*	
West	134 (46.2)
South	65 (22.4)
Northeast	42 (14.5)
Midwest	49 (16.9)
Annual Budget (median, IQR)	$70,398 ($9,013 – $196,950)
*Types of funding*	
Governmental funding	212 (73.1)
No governmental funding	78 (26.9)
*Number of different funding sources*	
0	15 (5.2)
1	119 (41.0)
2	53 (18.3)
3	53 (18.3)
> = 4	50 (17.2)
*Began telehealth services in 2020*	
Yes	87 (30.0)
No	203 (70.0)
*Telehealth intervention type*	
Medical intervention	67 (23.1)
Non-medical intervention	81 (27.9)
*Types of telehealth services (n = 87)*	
*Medical*	
HIV status counseling	31 (10.7)
HIV medical care	21 (7.2)
HCV status counseling	30 (10.3)
HCV medical care	22 (7.6)
Buprenorphine/Suboxone	49 (16.9)
*Non-medical*	
Wellbeing check-ins	44 (15.2)
Overdose prevention training	58 (20.0)
Health navigation/case management	55 (19.0)
Harm reduction counseling	63 (21.7)
Psychological counseling	26 (9.0)

*CBO* Community-based organization, *PHD* Public health department

In multivariable logistic regression models, CBO-SSPs had higher odds of having implemented medical (aOR = 4.69, 95% CI [1.96, 11.19]) and non-medical (aOR = 2.18, 95% CI [1.10, 4.31]) services via telehealth as compared to PHD-SSPs (Table 2). For medical interventions via telehealth, SSPs that received governmental funding (federal, state, or local) had higher odds of having implemented medical interventions via telehealth (aOR = 2.45, 95% CI [1.35, 4.47]) compared to programs who did not receive governmental funding. In addition, the Northeast Census region had higher odds of having implemented medical interventions via telehealth (aOR = 4.29, 95% CI [1.79, 10.31]) compared to the West Census region. For non-medical interventions, the Northeast (aOR = 6.67, 95% CI [2.28, 19.52]), the South (aOR = 3.90, 95% CI [1.28, 11.80]), and the Midwest (aOR = 3.37, 95% CI [1.13, 10.02]) had higher odds of having implemented non-medical telehealth services compared to the West.

**Table 2:** Association of SSP operational characteristics with availability of medical and non-medical telehealth-based health services in 2020

Characteristic	Medical interventions via telehealth*		Non-medical interventions via telehealth**	
	aOR	95% CI	aOR	95% CI
*Program type*				
CBO-SSP	**4.69**	**1.96, 11.19**	**2.18**	**1.10, 4.31**
PHD-SSP	REF	REF	REF	REF
*Census Region*				
Midwest	1.95	0.68, 5.57	**3.37**	**1.13, 10.02**
South	2.52	0.69, 9.20	**3.90**	**1.28, 11.80**
Northeast	**4.29**	**1.79, 10.31**	**6.67**	**2.28, 19.52**
West	REF	REF	REF	REF
*Funding Source*				
Governmental	**2.45**	**1.35, 4.47**	1.73	0.83, 3.62
No governmental	REF	REF	REF	REF

Bolded adjusted odds ratios represent *p* < 0.05

^*^Medical interventions included: HIV status counseling, HIV medical care, HCV status counseling, HCV medical care, and Buprenorphine/Suboxone

^**^Non-medical interventions included: wellbeing/check-ins, overdose prevention training, health navigation/case management, harm reduction counseling, and psychological counseling

*CBO* Community-based organization, *PHD* Public health department

## Discussion

We found that in the first year of the COVID-19 pandemic, 30% of SSPs newly implemented medical and/or non-medical services via telehealth. The recent rise of telehealth co-located at SSPs has expanded the diversity of services PWID can receive in this context with only 6% of SSPs offering telehealth-based services in 2019 [5] . One programmatic survey noted escalated telehealth delivery of primary care and mental health care across SSPs [5]. At the height of the COVID-19 pandemic, an SSP in New Haven, Connecticut harnessed telehealth in combination with bundled laboratory analyses to screen and treat PWID with HCV, resulting in 93.5% of PWID with sustained virologic response [25, 26]. These auspicious results, combined with our findings that CBO-SSPs were more likely to implement medical telehealth, present a new implementation strategy to bring high quality HIV and HCV care to traditionally marginalized communities.

Our results show that CBO-SSPs, presumably with fewer regulations than PHD-SSPs operating within governmental bureaucracies, had higher odds of having adopted telehealth for medical and non-medical services. However, programs that received governmental funding, perhaps a reflection of program maturity as well as government sanctioned operations [27], also had higher odds of having implemented telehealth for medical services compared to those without government funding. Our results show that SSPs operating with government funding, independent of the significant barriers of governmental institutional bureaucracy, were early adopters of telehealth services. This finding suggests that local, state, and federal governments should increase funding to SSPs, as it could facilitate provision of telehealth services to vulnerable and hard to reach populations.

As a region, the Northeast had the highest odds of having implemented telehealth, followed by the South. It is notable that the most commonly implemented telehealth services in 2020 were harm reduction counseling, overdose prevention training and health navigation/case management, which are all non-medical services that do not require a licensed clinician. It is possible that the scale of the opioid overdose mortality crisis contributed to increased priority of overdose education in telehealth implementation. While medical telehealth offered via SSPs is a new way to bring medical services to a community that has largely been left behind, non-medical telehealth is a new approach that could extend the reach of harm reduction into more communities. A non-medical telehealth harm reduction model could have significant potential to scale harm reduction services into more diverse locations until harm reduction organizations and SSPs are established in all geographic areas of need.

Early adoption of telehealth services in response to the pandemic could be a transformational change for SSPs, a historically limited resource setting [28]. As the traditional healthcare system continues to be an inhospitable environment for PWID due to pervasive stigma and numerous structural barriers [29, 30], SSPs have emerged as ideal venues for provision of comprehensive PWID-specific healthcare including buprenorphine, PrEP, HIV treatment, HCV treatment and primary care [25, 31–34]. SSPs have shown they can provide access to technology-based solutions for their participants [35–37] and have emerged as a potential one-stop shop for PWID healthcare needs.

Telehealth serves as a critical adaptation of the physician–patient encounter to deliver lifesaving treatments in low-barrier settings and at destigmatizing venues. Some well-resourced SSPs with clinicians on-site already provide a range of health services such as HIV/HCV testing [38], linkage to and/or on-site provision of substance use treatment [39, 40] , HIV care [31] , HCV treatment [41] , MOUD [32] , PrEP [42] , overdose prevention through naloxone distribution [43, wound care and general primary care [44 . Yet, this in-person model is more expensive and difficult to scale for all hours an SSP operates and in certain locations, such as rural areas. An increase in the use of telehealth, facilitated by equipment and Wi-Fi at the SSP to overcome the digital divide [45–47], could enable PWID who frequent these venues to have increased access to on-demand health services including primary care, infectious disease services, mental health services, etc. [48]. Large hybrid effectiveness-implementation trials [49] will be necessary to determine effective telehealth-based models at SSPs and to understand implementation considerations in these resource limited settings.

There are several potential limitations to this study that need to be considered when interpreting the results. First, this cross-sectional survey only describes use of telehealth in the first year of the pandemic. Follow-up assessments as part of the National Survey of Syringe Services Programs [49] will be essential to monitor trends in the use of telehealth at SSPs. Second, this survey does not include responses from all SSPs in the USA. While we achieved a 75% response rate of known SSPs operating throughout the USA, it is possible that smaller, less resourced programs were unable to participate in our survey and there could be other SSPs that we have yet to identify. However, the SSP database that we have developed in partnership with the North American Syringe Exchange Network is the most comprehensive database of SSPs in the USA. Finally, all data are self-reported and could be subject to social desirability bias, though the research team is well known to SSPs nationwide. Nonetheless, this important study highlights the ability of SSPs operating in collaboration with the traditional healthcare system to adapt expeditiously to a Public Health Emergency by providing remote, telehealth-based provision of services.

## Conclusion

CBO-SSPs and those that received some government funding had the highest odds of having implemented telehealth-based services in 2020 in response to the COVID-19 Public Health Emergency. Federal, state, and local governments must increase funding for SSPs, particularly non-governmental programs, to allow for SSPs to continue to adapt dynamically to the needs of PWID in the provision of medical and non-medical services. These low-barrier settings are ideal venues to deliver lifesaving services such as HIV care, PrEP, HCV treatment, and buprenorphine, leveraging their trust among PWID to improve health outcomes in this community during the new telehealth era.

## Data Availability

The datasets used and/or analyzed during the current study are available from Barrot H. Lambdin on reasonable request.

## Abbreviations

aOR: Adjusted odds ratios
CI: Confidence intervals
CBO-SSP: Community-based organization syringe services program
HCV: Hepatitis C virus
MOUD: Medication for opioid use disorder
NASEN: North American Syringe Exchange Network
OUD: Opioid use disorder
PHD-SSP: Public health department syringe services program
PWID: People who inject drugs
SSP: Syringe services program
SUD: Substance use disorder
USA: United States of America
